# Predicting severity of cerebral amyloid angiopathy neuropathology: A modeling approach using NACC and ROSMAP data

**DOI:** 10.1002/alz.70057

**Published:** 2025-10-07

**Authors:** Chenyin Chu, Liwei Ma, Yihan Wang, Andrew L. H. Huynh, Megan K. Dunstan, Xinran Cui, Colin L. Masters, Benjamin Goudey, Liang Jin, Yijun Pan

**Affiliations:** ^1^ School of Translational Medicine, Monash University Melbourne Victoria Australia; ^2^ The Florey Institute of Neuroscience and Mental Health Parkville Victoria Australia; ^3^ Department of Aged Care Austin Health Heidelberg Victoria Australia; ^4^ Department of Medicine Austin Health The University of Melbourne Heidelberg Victoria Australia; ^5^ Australian BioCommons The University of Melbourne North Melbourne Victoria Australia

**Keywords:** amyloid‐related imaging abnormalities, AutoScore algorithm, cerebral amyloid angiopathy, machine learning, risk stratification

## Abstract

**INTRODUCTION:**

Cerebral amyloid angiopathy (CAA) is associated with an increased risk of amyloid‐related imaging abnormalities (ARIA) in patients with Alzheimer's disease using anti‐amyloid beta (Aβ) monoclonal antibody drugs. Here, we developed a tool, CAA risk score (CAARS) to predict the severity of CAA neuropathology.

**METHODS:**

The National Alzheimer's Coordinating Center (NACC) data were used to develop the CAARS, which was then externally validated using the Religious Orders Study and Memory and Aging Project (ROSMAP) data.

**RESULTS:**

The CAARS‐4 model achieved a mean area under the receiver‐operating characteristic (ROC) curve (AUC‐ROC) of 0.71 (95% confidence interval [CI]: 0.69–0.72) and a Harrell's generalized C‐index of 0.69 (95% CI: 0.68–0.71) in the NACC cohort validation. It outperformed the baseline models, and the promising performance was validated on ROSMAP participants, demonstrating the robustness and generalizability of the model.

**DISCUSSION:**

CAARS has the potential to predict CAA severity; however, its clinical utility should be evaluated in follow‐up studies.

**Highlights:**

- Cerebral amyloid angiopathy (CAA) severity can only be confirmed postmortem.
- CAA is associated with an elevated risk of amyloid‐related imaging abnormalities (ARIA).
- The CAA risk score (CAARS) can stratify CAA risks in living patients.
- Hypertension and apolipoprotein E (*APOE*) ε4 are risk factors for CAA.
- The CAARS has the potential to predict risk of ARIA.

## 1 BACKGROUND

Cerebral amyloid angiopathy (CAA) is a cerebrovascular disorder characterized by the deposition of amyloid beta (Aβ) proteins in the walls of cerebral blood vessels.^1^ Diagnosing CAA remains challenging, with the updated Boston Criteria 2.0 offering a clinical framework for identifying probable or possible CAA based on the characteristic hemorrhagic and white matter changes visible on brain magnetic resonance imaging (MRI) in individuals 50 years of age or older, provided that alternative causes are excluded.^2^, ^3^ Definitive confirmation of CAA, however, requires postmortem brain examination, which remains the only method to accurately determine its severity, categorized as none, mild, moderate, or severe.^4^

CAA has garnered increasing attention from clinicians and dementia researchers due to its association with amyloid‐related imaging abnormalities (ARIA), a significant adverse effect observed in clinical trials of monoclonal antibody (MAB) therapies targeting Aβ in Alzheimer's disease (AD).^5^, ^6^ ARIA is understood to result from increased inflammation and permeability of the blood vessels in the brain. This can result in increased brain swelling or edema (ARIA‐E) or deposition of blood products in the brain and small microhemorrhages (ARIA‐H).^7^ Although often asymptomatic, some patients with ARIA can experience serious adverse effects, including confusion, headaches, nausea, seizures, dizziness, and rarely, death. Once established, the presence of ARIA is further associated with an increased risk of intracerebral hemorrhage (or macro‐hemorrhage) for patients with ARIA who later require antithrombotic, thrombolytic, or anticoagulation therapy for management of coronary artery disease, stroke, or atrial fibrillation.^8^ Considering the risks versus benefits associated with using MABs, clinicians may be hesitant to prescribe these new drugs to people with AD. Tools capable of assessing the severity of CAA and possibly predicting ARIA risk could facilitate informed risk–benefit discussions and enhance clinical decision‐making. In addition, predictive tools could be adapted to stratify ARIA risk, aiding in the personalization of MRI monitoring protocols during MAB treatment.

In this study, we developed and validated a machine learning model, CAA risk score (CAARS). This model is built on an AutoScore and AutoScore‐Ordinal algorithm, developed using data collected by National Alzheimer's Coordinating Center (NACC),^9^, ^10^, ^11^ and externally validated using data collected in the Religious Orders Study and Memory and Aging Project (ROSMAP).^12^

## 2 METHODS

### 2.1 Data sources and ethics

Data were collected from the NACC cohort for model development and validation. Established in 1999, the NACC is a comprehensive relational database comprising standardized clinical and neuropathological research data collected from Alzheimer's Disease Centers (ADCs) across the United States. Data collection was approved by institutional review boards at each ADC. Each ADC employed its own protocol to obtain informed consent from participants or, when participants were unable to provide consent, from their next of kin, carers, or legal guardians. Data from the NACC was requested via their official website: https://naccdata.org/. The Religious Orders Study (ROS) and The Memory and Aging Project (MAP) are longitudinal, epidemiologic clinical‐pathological studies of aging and dementia, recruiting participants without known dementia in the United States, started in 1994 and 1997, respectively. ROSMAP study was approved by the Rush University Medical Centre Institutional Review Board, and all participants provided informed consent. ROSMAP data was obtained from https://www.radc.rush.edu. The current study analyzes the de‐identified secondary data collected by NACC and ROSMAP, and informed consent or local ethical committee approval was therefore not required.

**RESEARCH IN CONTEXT**

1. **Systematic review**: The authors conducted a comprehensive literature review using PubMed, Google Scholar, and Web of Science to explore existing machine learning models to predict cerebral amyloid angiopathy (CAA) severity. Currently, no machine learning model is available to satisfy this need in living patients.
2. **Interpretation**: We developed and validated the CAA risk score (CAARS) to predict CAA neuropathological severity. The model was constructed using the AutoScore algorithm, which automates the development of interpretable clinical scoring models. CAARS achieved promising prediction performance for National Alzheimer's Coordinating Center (NACC) participants, which was reproducible on the Religious Orders Study/Memory and Aging Project (ROSMAP) participants. The machine learning model and epidemiology analysis indicated that apolipoprotein E (*APOE*) ε4 and hypertension are risk factors for CAA.
3. **Future directions**: CAARS holds the potential to predict risk of amyloid‐related imaging abnormalities (ARIA), and to inform antithrombotic use and predict intracerebral hemorrhage risk in CAA. Its clinical utility should be validated in future studies.

### 2.2 Participants and variables

We used the NACC data for model development. A total of 7064 NACC participants who had postmortem CAA neuropathology characterized (none, mild, moderate, severe) were identified from the dataset. During data pre‐processing, variables (potential predictors) with more than 10% missing data were excluded from analysis. The remaining variables are listed in eTable 1. Participants with missing values for any included variable were removed. This resulted in a final cohort of 4134 NACC participants for the study. Following the same procedure, we identified 1727 ROSMAP participants, and their data were used for external validation of the CAARS.

### 2.3 CAARS model construction

The predicted outcome in our study is the severity of CAA pathology defined by neuropathologists—classified as none, mild, moderate, or severe—making it well‐suited for modeling as an ordinal outcome. An alternative predicted outcome is the presence of CAA, classified as none versus mild/moderate/severe. The development of the CAARS leverages the AutoScore and AutoScore‐Ordinal algorithms and their accompanying R packages.^13^, ^14^, ^15^ An architecture framework has been prepared to illustrate the model construction process (eFigure 1).

The AutoScore and AutoScore‐Ordinal algorithms consist of six modules. (1) Feature ranking: Variables are ranked by their importance using a random forest algorithm specifically designed for binary/ordinal classification tasks. (2) Variable transformation: Continuous variables are converted into categorical variables to enhance interpretability and accommodate potential nonlinear relationships with the outcome. This approach, commonly used in medical research, minimizes the impact of outliers on model performance.^16^ (3) Score derivation: Variable weights are calculated using the cumulative link model, a robust regression method for ordinal outcome.^17^ These weights are then normalized and rescaled to produce scores for each variable. (4) Feature selection: A parsimony analysis incorporating 10‐fold cross‐validation is conducted to determine the optimal number of variables, ensuring a balance between model simplicity and performance. (5) The algorithm‐generated cutoff values for categorized variables are adjusted to align with standard clinical guidelines and norms to maintain clinical relevance. Categories with small sample sizes, which led to scores lacking clinical interpretation, were re‐evaluated. (6) Model evaluation: The finalized model is evaluated on an unseen dataset to assess its predictive performance.

For this study, the NACC dataset was split into a training set (70%) and a test set (30%). The training set was used for Modules 1–5, and the test set was used for Module 6. Additional details on the AutoScore and AutoScore‐Ordinal framework and data processing methods are provided in the Supplementary Methods. We developed CAARS‐4 categorizing CAA severity as none, mild, moderate, severe; and CAARS‐2 to identify individuals at risk of developing CAA (none vs mild/moderate/severe). Each model addresses a different range and extent of severity to provide flexibility in clinical applications. This multi‐model approach could possibly enhance clinical utility by providing tailored predictions for various stratification needs, paving the way for future research to determine the most effective risk assessment strategy.

### 2.4 CAARS model evaluation

For CAARS‐4, the model evaluation was conducted on the test set using two key metrics: the mean area under the receiver‐operating characteristic (ROC) curve (mean AUC‐ROC) and Harrell's generalized C‐index. For classification tasks involving *N* categories, the problem was divided into *N*−1 binary classification tasks, and the mean AUC‐ROC was calculated as the average AUC across these tasks.^18^ Harrell's generalized C‐index measures the proportion of concordant pairs—those in which the predicted rankings correspond with the observed outcomes, including instances of tied ranks—relative to all possible observation pairs. This metric is widely utilized to assess the performance of classification models.^19^, ^20^ For both metrics, a value of 0.5 signifies random performance, whereas a value of 1 reflects perfect prediction. The evaluation results were supplemented with bias‐corrected 95% bootstrap confidence intervals (CIs). Sensitivity and specificity were also calculated specifically for the CAARS‐2 models, as these models address binary classification.

The performance of the CAARS models was compared against several baseline models, including a proportional odds model with LASSO feature selection (POM‐1), a proportional odds model with stepwise feature selection (POM‐2), and a proportional odds model utilizing features selected from Modules 1 and 4 of CAARS (POM‐3). This comparison underscored the advantages of the CAARS‐4 models, which employ AutoScore‐Ordinal algorithms, over traditional ordinal outcome classification models. For binary classification, additional comparisons were made with logistic regression models that used random forest–based feature selection (logistic‐base). These models were chosen as baselines due to their common use in binary classification tasks.

### 2.5 External validation on ROSMAP data

To ensure the generalizability of the CAARS models, we conducted external validation using an independent cohort of 1727 participants from the ROSMAP. Due to differences between the two cohorts, only variables present in both were included. Although the AutoScore‐Ordinal algorithm identifies features based on random forest feature selection and parsimony analysis, it is still possible to manually select variables for multi‐cohort evaluations. To assess predictive accuracy, the CAARS model predictions were compared against clinical diagnoses. Performance was quantified using mean AUC‐ROC and Harrell's generalized C‐index, providing insights into the models’ discriminative ability and overall concordance with clinical outcomes.

### 2.6 Epidemiology analysis

A multivariable logistic regression model was employed to explore the targeted longitudinal associations between features selected for the CAARS and the risk of CAA. To avoid reverse causality between potential covariates and the risk of CAA, participants with a follow‐up period of less than 5 years were excluded. A total of 2056 NACC participants were included for epidemiology analysis. To reduce survival and time‐dependent biases, we adjusted for follow‐up duration. In addition, to address potential confounding bias, we adjusted for sex, age, body mass index (BMI), apolipoprotein E (*APOE*) ε4 carrier status, and blood pressure (systolic and diastolic). All tests were two‐sided, with statistical significance set at *p *< .05.[Table alz70057-tbl-0001]

### 2.7 Software and packages

All data preprocessing and analyses were performed using Python version 3.9, RStudio version 12.0+369, and Stata software (version 17.0). The developed model relies on the AutoScore package in the R 3.5.3 programming environment (R Foundation).^15^ This package facilitates the streamlined creation of point‐based clinical scoring models for outcome prediction, reducing the need for extensive manual input in tasks such as data processing, parameter tuning, and model optimization.

## 3 RESULTS

### 3.1 Participant characteristics

The CAARS model was developed using an AutoScore‐Ordinal algorithm, based on data from 4134 participants in the NACC cohort, which is then externally evaluated on 1727 ROSMAP participants. For the NACC participants, the mean baseline age was 75.31 years (SD = 10.01), with 43.7% *APOE* ε4 carriers. The cohort comprised 47.2% women, with an average education level of 15.68 years (SD = 2.86). At the time of death, neuropathological diagnoses of CAA were distributed as follows: 39.1% none, 30.6% mild, 19.5% moderate, and 11.8% severe. The ROSMAP participants had a mean baseline age of 80.10 years (SD = 7.00), with 25.8% *APOE* ε4 carriers. The cohort included 68.9% women and had an average education level of 16.29 years (SD = 3.58). At the time of death, neuropathological diagnoses of CAA were distributed as follows: 21.3% none, 41.0% mild, 23.0% moderate, and 14.7% severe. Detailed participant characteristics are provided in Table 1.

**TABLE 1 alz70057-tbl-0001:** Participant characteristics.

Features	NACC (*n* = 4134)	ROSMAP (*n* = 1727)
Age (years)	75.31 (10.01)	80.10 (7.00)
Sex	Female: 1952, Male: 2182	Female: 1190, Male: 537
Body mass index	26.44 (4.68)	26.77 (4.89)
Education (years)	15.68 (2.86)	16.29 (3.58)
*APOE* genotype	ε3/ε3: 1962; ε4/ε3: 1339; ε3/ε2: 347; ε4/ε4: 340; ε4/ε2: 126; ε2/ε2: 20	ε3/ε3: 1061; ε4/ε3: 382; ε3/ε2: 211; ε4/ε4: 30; ε4/ε2: 33; ε2/ε2: 10
Time after baseline (years)	5.07 (3.60)	8.95 (5.81)
Diastolic blood pressure (mmHg)	74.08 (10.46)	72.62 (10.75)
Systolic blood pressure (mmHg)	134.70 (18.66)	135.71 (17.81)
WAIS‐R	33.66 (14.47)	NA
Heart rate (per minute)	67.50 (10.64)	NA
Animal naming test^a^	14.84 (6.31)	NA
Logical memory test^b^	8.35 (5.53)	NA
Boston naming test (30 items)	24.00 (5.92)	NA
Geriatric Depression Scale	2.17 (2.44)	1.14 (1.56)
Mini‐Mental State Examination	25.49 (4.95)	27.60 (2.83)
CAA pathology^c^	0: 1616; 1: 1264; 2: 806; 3: 448	0: 367; 1: 708; 2: 398; 3: 254

*Notes*: Data are presented as mean (SD). All data were collected at baseline except neuropathology data (i.e., CAA pathology).

Abbreviations: ADCs, Alzheimer's Disease Centers; CAA, cerebral amyloid angiopathy; CAARS, CAA risk score; NA, not applicable; NACC, National Alzheimer's Coordinating Center; ROSMAP, Religious Orders Study and Memory and Aging Project. WAIS‐R, Wechsler Adult Intelligence Scale–Revised.

^a^ Total number of animals named in 60 s.

^b^ Total number of story units recalled from this current test administration.

^c^ Neuropathological evaluations were conducted by individual ADCs following their own protocols, which may vary but are in accordance with consensus guidelines. The results were entered into the NACC database using a standardized NACC form. The presence of CAA was detected using amyloid stains (e.g., Congo red, thioflavin‐S, or amyloid beta [Aβ] immunostaining), as determined by each ADC's protocol. CAA severity was graded semi‐quantitatively as none (0), mild (1), moderate (2), or severe (3). According to the NACC coding guidelines, severity was based on the neuropathologist's overall assessment of severity, rather than on the condition of an individual vessel.

### 3.2 Outcomes of CAARS‐4 model construction and evaluation

**FIGURE 1:**
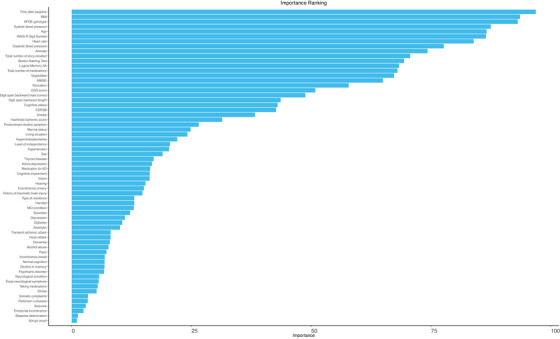
Feature ranking. This plot depicts the relative importance of features derived from random forest–based feature selection. The *x*‐axis quantifies the importance score of each feature, whereas the *y*‐axis lists the names of the analyzed features. Wider bars correspond to higher feature importance, highlighting predictors that contribute most significantly to the model's performance.

The AutoScore‐Ordinal algorithm employed a random forest method to rank variables by their importance in predicting CAA severity. As shown in Figure 1, time after baseline was the most important predictor, which is expected as aging is a risk factor for CAA.^21^ The *APOE* genotype is another important predictor, aligning with clinical evidence that the ε4 allele of *APOE* is a known risk factor for CAA.^22^ Moreover, BMI is also identified as a key predictor. Additional variables, including blood pressure, age at baseline, and education, are also highlighted as important, consistent with findings from clinical and epidemiological studies.^23^, ^24^

**FIGURE 2:**
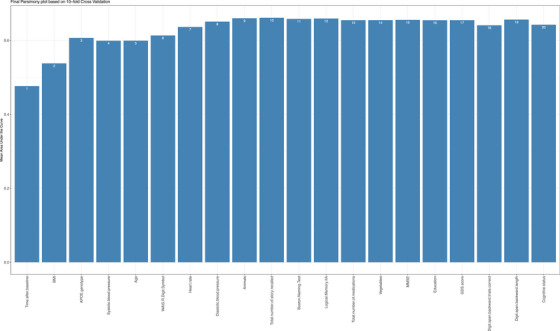
Parsimony plot for CAARS‐4. This plot illustrates the relationship between the number of predictors incorporated into the CAARS‐4 model and its performance, measured by the average mean AUC‐ROC values. The numerical labels on the bars indicate the cumulative count of predictors included at each step. Higher bars reflect superior model performance. AUC, area under the curve; CAARS, cerebral amyloid angiopathy (CAA) risk score; ROC, receiver‐operating characteristic.

To optimize the balance between complexity and performance in the CAARS‐4 model, a parsimony analysis was conducted to determine the optimal number of predictors. As shown in Figure 2, the mean AUC‐ROC increased rapidly when the model incorporated one to three features, rising from 0.47 to 0.60. However, when additional two predictors were included, the model's performance remained the same, and the performance then improved consistently as the number of predictors increased from 6 to 10, with the mean AUC‐ROC rising from 0.59 to 0.67. No further improvement in the mean AUC‐ROC was observed by including additional predictors. Therefore, the CAARS‐4 model employed 10 predictors, striking a balance between simplicity and predictive accuracy.

After identifying the most relevant variables, we applied a cumulative log‐link regression model to assign scores for each category or interval of the selected predictors (Table 2, left panel). The maximum CAARS score was set at 100. On the test set, the model achieved a mean AUC‐ROC of 0.70 (95% CI: 0.68–0.72] and Harrell's generalized C‐index of 0.67 (0.66–0.70) before fine‐tuning. To improve clinical utility, we refined the intervals for each feature as per their clinical norms. For example, BMI categories were adjusted to [0, 18.5), [18.5, 25), [25, 30), and  ≥30^25^; diastolic blood pressure intervals as <60, [60, 80), [80, 90), and ≥90;0 systolic blood pressure intervals as <120, [120, 150), and ≥150^26^; Wechsler Adult Intelligence Scale‐Revised (WAIS‐R) Digit Symbol test as <30, [30, 40), [40, 49), and ≥49.^27^ The revised scores for each feature following fine tuning are presented in Table 2.

**TABLE 2:** Score table for CAARS‐4 model.

Variable	Before fine‐tuning		After fine‐tuning	
	Interval	Score	Interval	Score
Time after baseline	<2.01	0	<2.01	0
	[2.01,8.16)	6	[2.01,8.16)	6
	≥8.16	7	≥8.16	8
Body mass index^a^	<29.9	3	<25	3
	[29.9,35.3)	0	[25,30)	2
	≥35.3	2	≥30	0
*APOE*	ε3/ε3	3	ε3/ε3	3
	ε3/ε4	20	ε3/ε4	22
	ε3/ε2	1	ε3/ε2	0
	ε4/ε4	41	ε4/ε4	45
	ε4/ε2	24	ε4/ε2	26
	ε2/ε2	0	ε2/ε2	0
Blood pressure (systolic)^a^	<108	4	<120	0
	[108,120)	0	[120,150)	4
	[120,150)	5	≥150	3
	≥167	4		
Age	<57	4	[57,67)	0
	[57,67)	0	[67,90)	7
	[67,83)	6	≥90	9
	[83,90)	7		
	≥90	9		
Wechsler Adult Intelligence Scale‐Revised^a^	<7	7	<30	4
	[7,22)	8	[30,49)	2
	[22,46)	2	≥49	0
	[46,57)	0		
	≥57	1		
Heart rate	<52	0	[52,60)	6
	[52,60)	6	≥60	4
	[60,76)	4		
	[76,84)	2		
	≥84	4		
Blood pressure (diastolic)^a^	<59	0	<60	0
	[58,66)	1	[60,90)	1
	[65,82)	2	≥90	2
	[82,90)	0		
	≥90	1		
Animal name test	<4	0	<4	0
	[4,10)	3	≥4	3
	[10,20)	4		
	[20,25)	2		
	≥25	4		
Total number of stories recalled	<3	14	<3	15
	[3,14)	8	[3,13)	9
	[14,18)	2	[13,18)	2
	≥18	0	≥18	0

Abbreviation: *APOE*, apolipoprotein E; CAARS, cerebral amyloid angiopathy risk score.

^a^ fine‐tuning based on clinical norms.

**FIGURE 3 alz70057-fig-0003:**
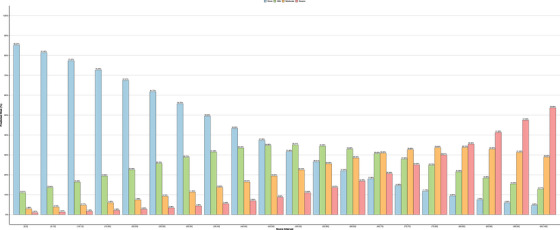
Score intervals and risk probability for CAARS‐4. The *x*‐axis represents CAARS score intervals, and the *y*‐axis shows the probability of CAA severity, represented by color‐coded bars (none: blue, mild: green, moderate: yellow, severe: pink). As the score increases, the probability of more severe CAA also rises. CAARS, cerebral amyloid angiopathy (CAA) risk score.

After fine‐tuning, the CAARS‐4 model retained comparable performance on the test set, with a mean AUC‐ROC of 0.71 (95% CI: 0.69–0.72) and Harrell's generalized C‐index of 0.69 (0.68–0.71). Although fine‐tuning did not affect the model performance, the fine‐tuned intervals based on clinical norms offer a more meaningful and interpretable scoring system compared to the quantile‐based intervals generated by the AutoScore algorithm. We subsequently mapped the scores to CAA severity: none (0–50), mild (50–65), moderate (65–80), and severe (80–100). A risk plot (Figure 3) illustrated the relationship between score intervals and the probability of CAA severity, revealing a clear trend of increasing severity with higher scores. These findings underscore the ability if the CAARS‐4 model to effectively stratify the severity of the condition.

### 3.3 Outcomes of CAARS‐2 model construction and evaluation

The CAARS‐2 model was developed using the AutoScore algorithm, which was designed to predict the binary classification of CAA (none vs mild/moderate/severe). The importance ranking of the predictors is presented in eFigure 2, which was highly consistent with those of the CAARS‐4, with minor differences in importance values. Parsimony analysis for CAARS‐2 is presented in eFigure 3, from which we determined that nine predictors were required to achieve the best model performance. The fine‐tuned score table is provided as eTable 2. The CAARS‐2 model achieved an AUC‐ROC of 0.71 (95% CI: 0.67–0.73), sensitivity of 0.61 (95% CI: 0.56–0.74), and specificity of 0.79 (95% CI: 0.75–0.83) at a threshold of 55.

### 3.4 Comparison between the baseline models and CAARS

The comparisons between the two CAARS models and their respective baseline models are summarized in Table 3A. The performance of CAARS‐4 exceeded that of POM‐1 (mean AUC‐ROC: 0.64 [95% CI: 0.61–0.66]; Harrell's generalized C‐index: 0.60 [95% CI, 0.57–0.62]), POM‐2 (mean AUC‐ROC: 0.66 [95% CI: 0.63–0.69]; Harrell's generalized C‐index: 0.61 [95% CI: 0.59–0.63]), and POM‐3 (mean AUC‐ROC: 0.67 [95% CI: 0.64–0.70]; Harrell's generalized C‐index: 0.63 [95% CI: 0.61–0.65]). Similarly, CAARS‐2 outperformed the logistic‐base model (AUC‐ROC: 0.63 [95% CI 0.60–0.66]; sensitivity: 0.21 [95% CI: 0.12–0.34]; specificity: 0.83 [95% CI: 0.76–0.88]). Overall, the CAARS models demonstrated superior performance compared to their baseline models.

**TABLE 3:** Model performance evaluation.

Model	Mean AUC‐ROC	Generalized C‐index	AUC‐ROC	Specificity	Sensitivity
**A: Comparison between CAARS models and baseline models**					
CAARS‐4	0.71 [95% CI: 0.69–0.72]	0.69 [95% CI, 0.68–0.71]	NA	NA	NA
POM‐1	0.64 (95% CI: 0.61–0.66)	0.60 (95% CI, 0.57–0.62)	NA	NA	NA
POM‐2	0.66 (95% CI: 0.63–0.69)	0.61 (95% CI, 0.59–0.63)	NA	NA	NA
POM‐3	0.67 (95% CI: 0.64–0.70)	0.63 (95% CI, 0.61–0.65)	NA	NA	NA
CAAS‐2	NA	NA	0.71 (95% CI: 0.67–0.73)	0.79 (95% CI: 0.75—0.83)	0.61 (95% CI: 0.56–0.74)
Logistic‐all	NA	NA	0.62 (95% CI: 0.58–0.65)	0.74 (95% CI: 0.67–0.75)	0.43 (95% CI: 0.38–0.50)
Logistic‐selected	NA	NA	0.63 (95% CI: 0.60–0.66)	0.83 (95% CI: 0.76–0.88)	0.21 (95% CI: 0.12–0.34)
**B: External evaluation of CAARS models**					
CAARS‐4^a^ (NACC)	0.70 (95% CI: 0.68‐0.72)	0.68 (95%CI: 0.67–0.71)	NA	NA	NA
CAARS‐4^a^ (ROSMAP)	0.69 (95% CI: 0.68–0.71)	0.68 (95% CI: 0.65–0.70)	NA	NA	NA
CAARS‐2^a^ (NACC)	NA	NA	0.71 (95% CI: 0.69–0.74)	0.82 (95% CI: 0.78–0.85)	0.60 (95% CI: 0.56–0.63)
CAARS‐2^a^ (ROSMAP)	NA	NA	0.70 (95% CI: 0.66–0.73)	0.70 (95% CI: 0.68–0.72)	0.58 (95% CI: 0.53–0.64)

*Notes*: Sky blue = classification of none, mild, moderate, severe. Purple = binary classification of none versus mild/moderate/severe. The AUC‐ROC of each CAARS model was compared to the baseline models using the Wilcoxon signed‐rank test, and the *p*‐value for each comparison was annotated for the corresponding baseline.

Abbreviations: AUC, area under the curve; CAARS, cerebral amyloid angiopathy risk score; NA, not applicable; NACC, National Alzheimer's Coordinating Center; POM, proportional odds model; ROC, receiver‐operating characteristic; ROSMAP, Religious Orders Study and Memory and Aging Project.

^a^ Model using predictors available in both NACC and ROSMAP datasets.

### 3.5 External evaluation results

We used the ROSMAP cohort for the external evaluation of CAARS; however, the predictors recorded by ROSMAP and NACC do not align perfectly. For CAARS‐4, the WAIS‐R Digit Symbol test, the total number of animals named in 60 s, the total number of story units recalled, and heart rate were not available for ROSMAP participants. As a result, these were replaced by the Mini‐Mental Status Examination (MMSE), Geriatric Depression Scale (GDS) score, and years of education, which are the next highest‐ranked predictors. For CAARS‐2, the external evaluation employed nine predictors, replacing WAIS‐R Digit Symbol test, heart rate, and the total number of animals named in 60 s by MMSE score, GDS score, and years of education. The model performance using ROSMAP data was comparable to that using NACC data (Table 3B). Overall, the external evaluation demonstrated that the CAARS can achieve good performance across independent datasets.

### 3.6 Epidemiology analysis

We performed epidemiology analysis to justify some high importance predictors determined by CAARS. As shown in Table 4, after adjusting for potential covariates, a positive *APOE* ε4 carrier status was significantly associated with increased odds of CAA across all severity levels (mild: odds ratio [OR] 2.752; moderate: OR 4.501; severe: OR 4.491) compared to the reference group, with an increase in the strength of association as CAA severity increased. In contrast, a positive *APOE* ε2 carrier status was significantly associated with a decreased odds of mild/moderate CAA (mild: OR 0.602; moderate: OR 0.593) compared to the reference group. In addition, participants at hypertension stage 1 (systolic 130–139 mmHg) had higher odds of CAA (mild: OR 1.442; moderate: OR 1.513) compared to the reference group. A similar trend was observed for participants at hypertension stage 2 (systolic ≥140 mmHg), where the odds of moderate CAA were also increased (OR 1.504). For participants with elevated diastolic blood pressure, significantly increased odds of mild CAA were found (OR 3.115) compared to the reference group. Of interest, participants at hypertension stage 2 (diastolic ≥90 mmHg) were found to have significantly decreased odds of CAA (OR 0.400).

**TABLE 4 alz70057-tbl-0004:** Longitudinal associations between *APOE* ε4 carrier status, BMI, blood pressure, and the risk of CAA.

Measures	OR [95% CI], *p*‐value (*n *= 2056)		
	Mild CAA	Moderate CAA	Severe CAA
***APOE* ε4 carrier status**	**Reference: without ε4**		
	**2.752 [2.180–3.474], *p *< .001**	**4.501 [3.483–5.816], *p *< .001**	**4.491 [3.283–6.144], *p *< .001**
***APOE* ε2 carrier status**	**Reference: without ε2**		
	**0.602 [0.434–0.836], *p *< .001**	**0.593 [0.410–0.859], *p *< .001**	0.669 [0.424–1.055], *p *= .008
**BMI** ^a^	**Reference: normal**		
Underweight	1.255 [0.463–3.404], *p = *.66	0.521 [0.131–2.075], *p *= .36	1.918 [0.618–;5.957], *p *= .26
Overweight	0.993 [0.776–1.271], *p = *.95	1.028 [0.782–1.352], *p *= .84	0.962 [0.690–1.342], *p *= .82
Obesity stage 1	0.961 [0.695–1.329], *p = *.81	0.834 [0.574–1.212], *p *= .34	0.649 [0.397–1.061], *p *= .09
Obesity stage 2	0.953 [0.565–1.607], *p = *.86	0.862 [0.474–1.568], *p *= .63	1.019 [0.500–;2.074], *p *= .96
**Blood pressure (systolic)** ^b^	**Reference: normal**		
Elevated	0.478 [0.186–1.232], *p *= .13	1.117 [0.443–2.816], *p *= .82	0.564 [0.147–2.164], *p *= .26
Hypertension stage 1	**1.442 [1.047–1.987], *p *= .03**	**1.513 [1.059–2.160], *p *= .02**	0.966 [0.633–1.473], *p *= .94
Hypertension stage 2	1.332 [0.981–1.808], *p *= .07	**1.504 [1.073–2.110], *p *= .02**	0.936 [0.628–1.395], *p *= .75
**Blood pressure (diastolic)** ^c^	**Reference: normal**		
Elevated	**3.115 [1.185–8.188], *p *= .02**	1.122 [0.431–2.916], *p *= .81	1.378 [0.343–5.529], *p *= .65
Hypertension stage 1	1.003 [0.717–1.401], *p *= .99	0.729 [0.496–1.071], *p *= .11	0.631 [0.377–1.055], *p *= .08
Hypertension stage 2	1.034 [0.619–1.725], *p *= .90	**0.400 [0.203–0.785], *p *= .008**	0.865 [0.425–1.761], *p *= .69

Abbreviation: CAARS, cerebral amyloid angiopathy risk score. CAA, cerebral amyloid angiopathy. *APOE*, apolipoprotein E. OR, odds ratio.

Bold values represent statistically significant differences at *p* < 0.05.

^a^ BMI: Body mass index, categorized as underweight (<18.5), normal [18.5–25), overweight [25–30), obesity stage 1 [30–35), and obesity stage 2 (>35).

^b^ Blood pressure (systolic): normal (≤120 mmHg), elevated (120–129 mmHg), hypertension stage 1 (130–139 mmHg), and hypertension stage 2 (≥140 mmHg).

^c^ Blood pressure (diastolic): normal (≤80 mmHg), elevated (≤80 mmHg, with; systolic being 120–129 mmHg), hypertension stage 1 (80–89 mmHg), and hypertension stage 2 (≥90 mmHg).

## 4 DISCUSSION

Developing models to predict the severity of CAA has gained increasing interest from AD researchers and clinicians. These models could potentially be used to inform antithrombotic use in people with CAA and may offer the possibility to predict intracerebral hemorrhage risk if the predicted CAA severity can be evaluated against clinical outcomes. In addition, given the possible link between CAA and ARIA risk from receiving MABs,^28^, ^29^, ^30^ tools to predict CAA may be used for ARIA risk stratification.

Our team previously developed the Florey Cerebral Amyloid Angiopathy Score (FCAAS) model and conducted an epidemiological study to investigate the risk factors associated with CAA pathology.^24^, ^31^ Using ROSMAP data, the FCAAS model predicts CAA severity based on postmortem neuropathological data (amyloid plaque density and tau tangle density), as well as clinical data collected at baseline. Although being the first model to predict CAA, the immediate clinical utility of FCAAS may be limited due to the absence of widely accepted biomarkers that are strongly correlated with pre‐mortem CAA neuropathology. In this study we address this issue by utilizing predictors that can be measured in living patients. The new model CAARS was developed using NACC data and externally validated using ROSMAP data to ensure generalizability.[Fig alz70057-fig-0003]

The CAARS models exhibited strong predictive performance, consistently outperforming baseline models (Table 3A).[Table alz70057-tbl-0004] This underscores the effectiveness of the AutoScore algorithm for ordinal and binary classification tasks, particularly in comparison to conventional methods used in the baseline models. We employed multi‐cohort validation in the current study to evaluate the performance of the CAARS‐4. CAARS‐4 was developed and internally evaluated using the NACC dataset, with an external evaluation performed on the ROSMAP dataset. The external evaluation demonstrated a strong performance of the CAARS‐4, achieving a mean AUC‐ROC of 0.68 (95% CI: 0.67–0.70) and Harrell's generalized C‐index of 0.68 (95% CI: 0.67–0.69), comparable to its performance on the NACC participants (Table 3B). We have also performed external evaluation for the CAARS‐2, which also demonstrated a robust performance of the model. Of interest, despite some differences in demographics between NACC and ROSMAP, the external evaluation performance of the CAARS model remains strong. This is possibly due to age being the only demographic feature selected. Overall, this highlighted the robustness of the model and its generalizability.

Of note, the WAIS‐R Digit Symbol test, the total number of animals named in 60 s, and the total number of story units recalled are neuropsychological tests that are not commonly used in clinical practice. Clinicians in memory clinics often face time constraints during clinical appointments. Some cognitive assessments, such as the WAIS‐R Digit Symbol test, require significant time, training, and the involvement of a neuropsychologist for accurate assessment. This can limit the clinical applicability of these tests, especially in settings with time and resource constraints, such as rural and remote areas where access to neuropsychologists could be limited. We have demonstrated the feasibility of replacing these predictors with alternative predictors—MMSE, GDS score, and years of education—that are routinely collected in clinical settings. Despite this substitution, the performance of the CAARS model remains robust. This adaptability underscores the advantage of using the AutoScore and AutoScore‐Ordinal algorithms in developing CAARS, enhancing the model's potential clinical utility in practice.

We noted some interesting findings from the generated scoring tables (Table 2, eTable 2). It is evident that predictors such as *APOE* variants (ε4/ε2, ε4/ε3, ε4/ε4, and ε2/ε2), time after baseline, BMI, and age contribute significantly to the CAARS score. Notably, the *APOE* ε4/ε4 variant received the highest score among all *APOE* genotypes, consistent with its strong association with increased Aβ accumulation.^32^ Although the *APOE* ε2 variant appears to be protective against CAA, it remains unclear whether this protective effect is related to *APOE* ε2’s known association with reduced AD risk.^33^ This was re‐illustrated by our epidemiology findings, where *APOE* ε4 was significantly associated with a higher odds of CAA and *APOE* ε2 was significantly associated with lower odds of CAA. It is interesting to note that the protective effect of *APOE* ε2 became invisible in the presence of *APOE* ε4, which could be explored in future studies. As illustrated in the score table, hypertension (stage 1) and hypertension (stage 2) contribute to CAA risk. This is well supported by our epidemiological data, showing that hypertension was associated with varying degrees of increased CAA risk. Unexpectedly, hypertension stage 2 (diastolic) was found to be significantly negatively associated with moderate CAA risk in the epidemiological analysis, which conflicted with the CAARS score table. This could possibly be explained by the small sample size (*n *= 23), limiting the statistical power to uncover the true association. Overall, we demonstrated that the CAARS is evidence based and clinically relevant for predicting CAA severity.

One of the key strengths of the current study is that we have addressed the “black box” issue associated with machine learning models. The term “black box” refers to many machine learning models, especially complex ones, operating in a way that makes it difficult to understand how they arrive at their decisions or predictions. In our study, the AutoScore algorithm generated a score table, which enhanced transparency of CAARS models by providing clear and clinically interpretable results. This would allow clinicians and researchers to better understand the model's predictions, thereby fostering trust in its outputs. In addition, we validated the model further by verifying it with epidemiological analysis, ensuring that the score table is supported by findings from more traditional methods. Some of the identified predictors for CAA appeared to be linked to dementia, such as MMSE score, GDS score, and years of education. It must be noted that these predictors are not specific to dementia but rather are better studied in the context of dementia. Further epidemiological studies could explore the relationship between these predictors and CAA pathology. We encourage machine learning experts to collaborate with clinical epidemiologists in the development of future models.

The current study is not without limitations. Although the model was developed and internally validated on the NACC dataset and externally evaluated on the ROSMAP cohort, both datasets were collected in the United States, which limits the generalizability of the findings to middle‐ or low‐income countries. Further evaluation of the CAARS should be conducted using data from other regions of the world. However, this could be challenging, as CAA neuropathology is not commonly assessed in cohort studies of aging and dementia. We recommend that all existing cohort studies and new studies consider collecting these important data. The NACC and ROSMAP studies primarily recruited White participants, with very limited African American and Asian participants. Ethnicity likely plays an important role in the pathogenesis and progression of many diseases. Future studies should aim to incorporate data from non‐White participants to enhance the global applicability and relevance of the findings. Last but not least, due to data availability constraints, the development of the CAARS model did not include biomarkers and imaging data, despite their potential to enhance the model's performance. This limitation should be addressed in future studies.

In conclusion, CAARS has been developed to predict the severity of CAA neuropathology using data that can be collected from living patients. The score‐based risk prediction system employed by the CAARS is more likely to be accepted by clinicians compared to using other machine learning models. Further studies should be conducted to expand the current work, and clinical evaluation will be required before this tool can be translated into clinical practice.

## CONFLICT OF INTEREST STATEMENT

The authors have no conflicts to report. Author disclosures are available in the Supporting Information.

## CONSENT STATEMENT

Each Alzheimer's Disease Center (ADC) that contributed data to the National Alzheimer's Coordinating Center (NACC) employed its own protocol to obtain informed consent from participants or, when participants were unable to provide consent, from their next of kin, caregivers, or legal guardians. Written informed consent was obtained from all participants by Rush University. All participants have consented to allow secondary analysis of the de‐identified data.

## Supporting information



Supporting Information

Supporting Information
