# Supplementary material for: Predicting severity of cerebral amyloid angiopathy neuropathology: A modeling approach using NACC and ROSMAP data
Source: Alzheimers Dement. 2025 Oct 7;21(10):e70057. doi: 10.1002/alz.70057 (PMC12504064; doi:10.1002/alz.70057)
Supplement: Supplementary file 1 — Supporting Information [file ALZ-21-e70057-s002.docx]

**Supplemental Material - Table of Content**

**(A)** **Supplementary methods**

Module 1: Feature Ranking

Module 2: Variable Transformation

Module 3: Score Derivation

Module 4: Model Selection and Optimization

Module 5: Fine-Tuning Cutoff Points

Module 6: Performance Evaluation

Data handling

**(B) eFigures**

eFigure 1: Architecture of CAARS.

eFigure 2: Feature ranking for the CAARS-2 model.

eFigure 3: Parsimony analysis of the CAARS-2 model.

**(C) eTables**

eTable 1: Variables included in this study

eTable 2: Fine-tuning score table for CAARS-2.

**(A) Supplementary methods**

The auto-score framework consists of six modules. For modules 1 to 5, data from 70% of the NACC participants (2894, training set) were used, while for module 6, data from the remaining 30% NACC participants (1240, test set) were utilized. The training/test data split was randomly performed ten times. For each training set, 10-fold cross-validation was performed for Modules 1-5. Steps of data handling are also provided here to ensure results reproducibility.

**Module 1: Feature Ranking**
Random Forest (RF), an ensemble machine learning algorithm, was utilized to identify the most significant features for score generation. RF integrates multiple decision trees, with each tree developed using a classification or regression tree methodology. The final output was based on the combined results of all trees, providing RF with strong resistance to overfitting. For classification tasks, the Gini index was applied to find optimal splits, minimizing the likelihood of misclassification.

**Module 2: Variable Transformation**

Following feature selection, the chosen variables undergo preprocessing and transformation. In this framework, variables with more than ten categories were treated as continuous. Continuous variables were converted into categorical ones to better model nonlinear effects, with a predefined maximum of five categories for practicality. For this study, continuous variables were divided into four intervals at the 0th, 25th, 50th, 75th, and 100th percentiles of their total values. Adjacent intervals assigned the same score (in Module 3) were automatically merged for simplicity.

**Module 3: Score Derivation**

Variable weights were calculated using the cumulative link model with a logit link function, a common method for ordinal outcomes. Let ***Y*** represent the ordinal outcome with ***J*** categories (1 to ***J***), and ***X*** denotes the predictor variables. The formula for the logit of cumulative probabilities is:

$$P\left( \boldsymbol{Y}\leq j | \boldsymbol{X} \right)=\log\left( \frac{p_{j}}{1-p_{j}} \right)=\alpha_{j}-\boldsymbol{X}^{T}\boldsymbol{\beta}$$

where$\alpha_{j}$is the category-specific intercept and $\boldsymbol{\beta}$ represents the regression coefficients. To simplify interpretation, the model is adjusted to ensure all $\boldsymbol{\beta}$ values are positive. Coefficients are normalized relative to the smallest $\boldsymbol{\beta}$, and scores for each category are calculated as $\beta_{j}\left( score \right)=round\left( \frac{\beta_{j}}{\beta_{low}} \right)$. These scores form a table, with the total score determined by summing category points.

**Module 4: Model Selection and Optimization**

The model was optimized for simplicity and predictive accuracy. A parsimony plot was used to balance these objectives, identifying the optimal number of features where performance improvements plateau.

**Module 5: Fine-Tuning Cutoff Points**

Continuous variable cutoffs generated in Module 2 were refined for clinical relevance by merging, rounding, or adjusting intervals to align with medical norms/guidelines. After these adjustments, Modules 2 and 3 were rerun to ensure the final model remained robust and clinically meaningful.

**Module 6: Performance Evaluation**

The model's predictive accuracy was evaluated using mean ROC curve analysis and Harrell’s generalized c-index. These metrics assess how well predictions rank against observed outcomes, including tied ranks, providing a comprehensive performance evaluation on unseen test data.

**Data Handling**

Step 1: Obtain the NACC dataset (https://naccdata.org/) and the ROSMAP dataset (https://www.rushu.rush.edu/) through formal application procedures.

Step 2: Review the datasets and select participants. Variables with more than 10% missing data were excluded from the analysis. Participants with complete records of the remaining variables were included for model development. The data was then split into a training set (70%) and a test set (30%).

Step 3: Use RStudio (version 12.0+369) to run the six modules of the auto-score-ordinal algorithm to generate the CAARS score table to predict CAA severity. Ensure that the auto-score-ordinal package ([nliulab/AutoScore-Ordinal (github.com)](https://github.com/nliulab/AutoScore-Ordinal) ) is installed in your R programming environment.

Step 4: Apply the CAARS score table to predict CAA severity on the test set and for external evaluation. The flowchart illustrating this process is presented in **eFigure 1**.

**(B) eFigures**


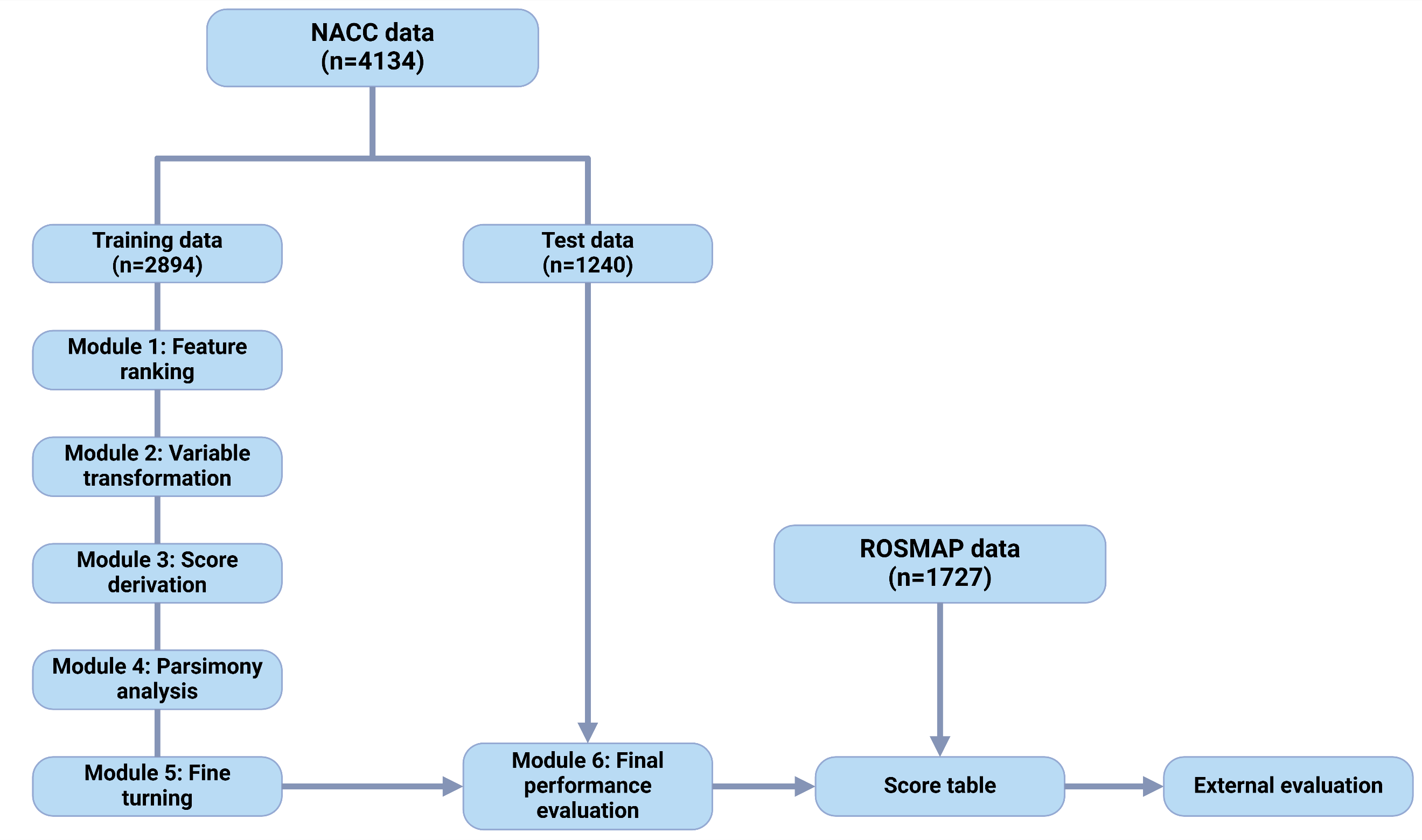


**eFigure 1: Architecture of CAARS.** The left panel illustrates the development and validation process of the CAARS model using NACC data, and the right panel presents its external evaluation using the ROSMAP data.


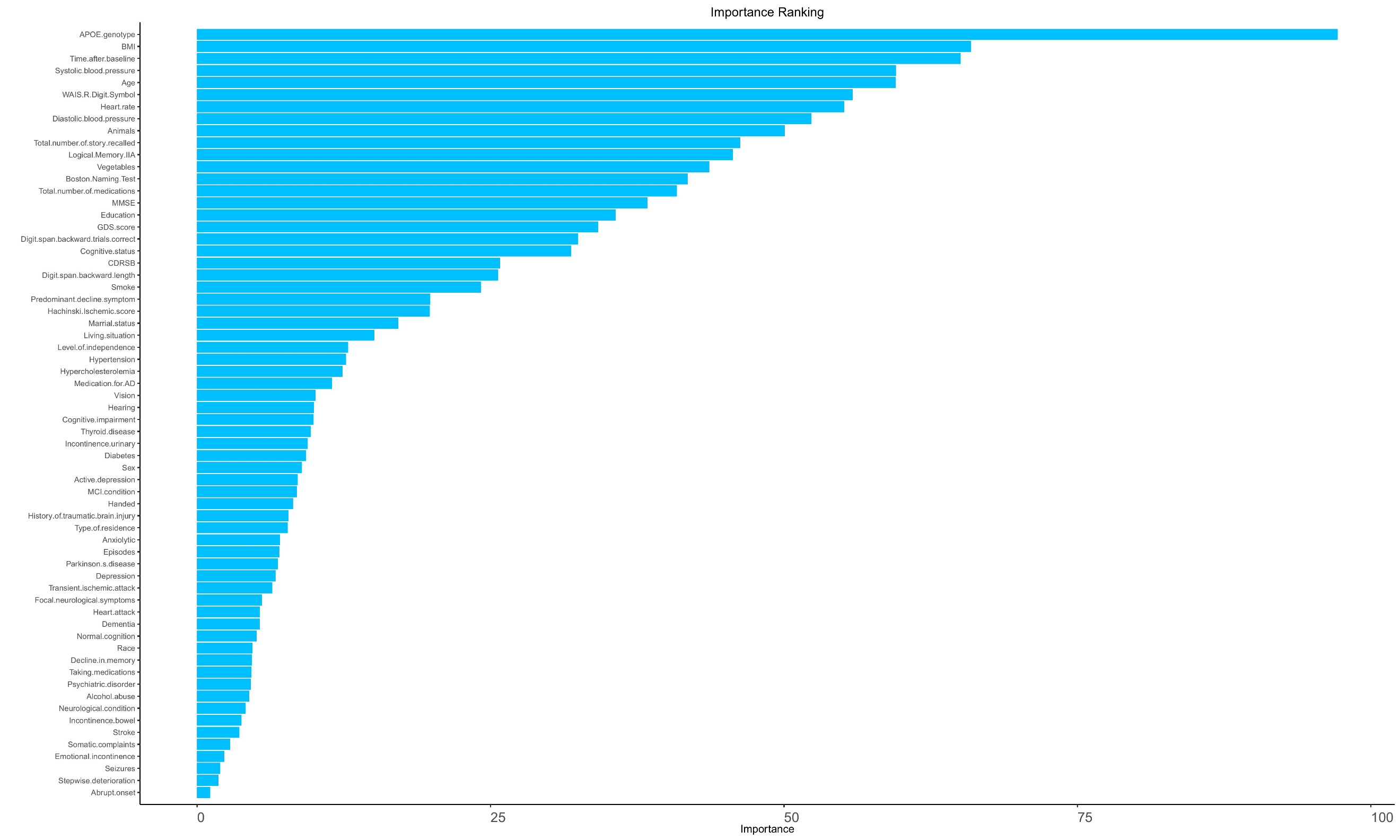


**eFigure 2: Feature ranking for the CAARS-2 model.** The x-axis represents the importance of features as determined by random forest feature selection, while the y-axis lists the names of all features included in the study. Wider bars indicate higher importance.

**
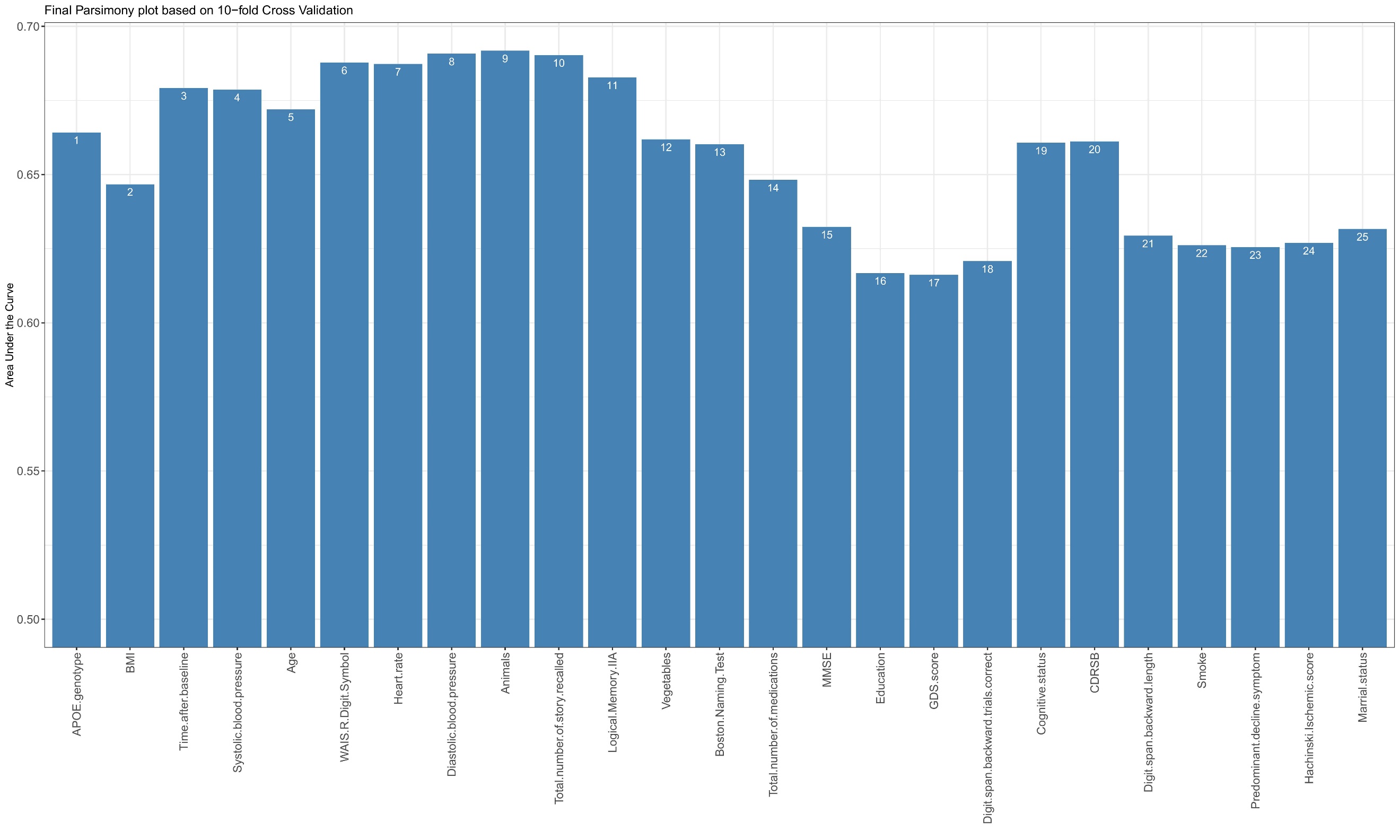
**

**eFigure 3: Parsimony analysis of the CAARS-2 model.** This plot shows the average mean AUC-ROC values as the number of variables included in the CAARS-2 model increases. The number annotated on each bar indicates the cumulative number of predictors used. Taller bars correspond to better model performance.

**(C) eTables**

**eTable 1: Variables included in the present study**

| APOE genotype | Abrupt onset | Active depression |
| --- | --- | --- |
| Age | Alcohol abuse | Animals |
| Anxiolytic | BMI | Boston Naming Test |
| CAA pathology | CDRSB | Cognitive impairment |
| Cognitive status | Decline in memory | Dementia |
| Depression | Diabetes | Diastolic blood pressure |
| Digit span backward length | Digit span backward trials correct | Education |
| Emotional incontinence | Episodes | Focal neurological symptoms |
| GDS score | Hachinski Ischemic score | Handed |
| Hearing | Heart attack | Heart rate |
| History of traumatic brain injury | Hypercholesterolemia | Hypertension |
| Incontinence bowel | Incontinence urinary | Level of independence |
| Living situation | Logical Memory IIA | MCI condition |
| MMSE | Marrial status | Medication for AD |
| Neurological condition | Normal cognition | Parkinson disease |
| Predominant decline symptom | Psychiatric disorder | Race |
| Seizures | Sex | Smoke |
| Somatic complaints | Stepwise deterioration | Stroke |
| Systolic blood pressure | Taking medications | Thyroid disease |
| Time to event | Total number of stories recalled | Total number of medications |
| Transient ischemic attack | Type of residence | Vegetables |
| Vision | WAIS R Digit Symbol |  |

Note: The explanation of each variable used in this study can be found at: [Uniform Data Set version 3 | National Alzheimer's Coordinating Center](https://naccdata.org/data-collection/forms-documentation/uds-3).

**eTable 2: Fine-tuning score table for CAARS-2.**

| Variable | Interval | Score |
| --- | --- | --- |
| APOE | ε3/ε3 | 9 |
|  | ε3/ε4 | 33 |
|  | ε3/ε2 | 1 |
|  | ε4/ε4 | 57 |
|  | ε4/ε2 | 30 |
|  | ε2/ε2 | 0 |
| Body mass index* | <30 | 3 |
|  | >=30 | 0 |
| Time after baseline | <2.24 | 0 |
|  | [2.24,4.74) | 4 |
|  | >=4.74 | 6 |
| Blood pressure  (systolic)* | <120 | 0 |
|  | [120,150) | 3 |
|  | >=150 | 4 |
| Age | <69 | 0 |
|  | [69,82) | 4 |
|  | >=82 | 7 |
| Wechsler Adult Intelligence  Scale-Revised* | <40 | 4 |
|  | [40,49) | 3 |
|  | >=49 | 0 |
| Heart rate | <60 | 6 |
|  | [60,67) | 4 |
|  | [67,73) | 3 |
|  | >=73 | 0 |
| Blood pressure  (diastolic)* | <60 | 0 |
|  | [60,90) | 6 |
|  | >=90 | 9 |
| Animal name test | <19 | 3 |
|  | >=19 | 0 |

* fine-tuning according to clinical norms
